# Supplementary material for: MiCas9 increases large size gene knock-in rates and reduces undesirable on-target and off-target indel edits
Source: Nat Commun. 2020 Nov 27;11:6082. doi: 10.1038/s41467-020-19842-2 (PMC7695827; doi:10.1038/s41467-020-19842-2)
Supplement: Supplementary file 1 — Supplementary Information [file 41467_2020_19842_MOESM1_ESM.pdf]

Supplementary Figure 1. MiCas9 design and production. (a) spCas9 (left) and miCas9 (right) expressing plasmid maps. (b) spCas9 and miCas9 protein expression in transfected Ad293 cells detected by Western blotting. (c) Representative gel for in-house production of Cas9 proteins. (d) In vitro digestion results by using miCas9 and spCas9 RNPs targeting human CDCP, CFTR and C3 genes.

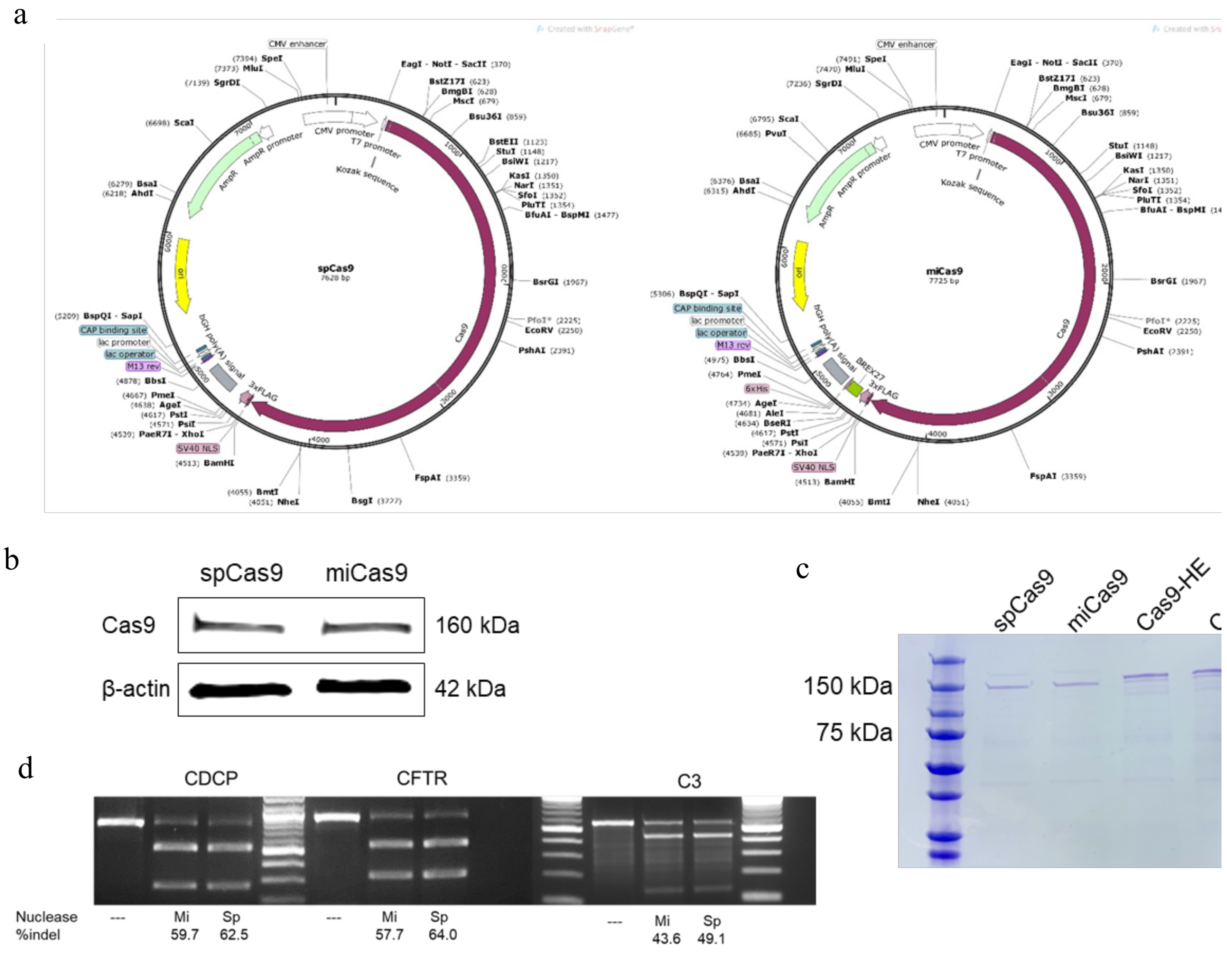

Supplementary Figure 2. **Application of miCas9 in ds-KI experiments**. (a) Illustration of the GFP expressing ds-DNA donor templates for large fragment knock in experiments. (b) Illustration of primer sets used in nest PCR (F1 and R2) to assess non-precise integration at the left junction (F1 and R1) and the right junction (F2 and R2) in the AAVS1 ds-KI experiment using sg-AAVS1 and GFP-donor-1k-AAVS1. (c) Summary of non-precise integration events determined by deep-seq at the junctions in the AAVS1 ds-KI experiment using sg-AAVS1 and GFP-donor-1k-AAVS1. (d) Percentage of GFP^{+} cells after GFP ds-KI at the AAVS1 using ds-DNA donor templates of different sizes (1K, 2K or 3K). SA: splicing acceptor. Sp: spCas9. Mi: miCas9. In (d), three independent experiments were performed for each condition. Data are presented as mean ± standard error of means (SEM). Unpaired t test (two-tailed) was used to compare data using GraphPad Prism 8 software (GraphPad Software, Inc., San Diego, CA).

a

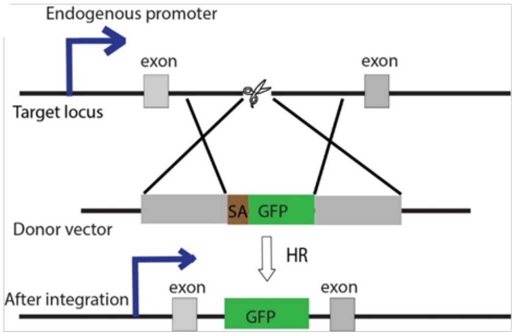

b

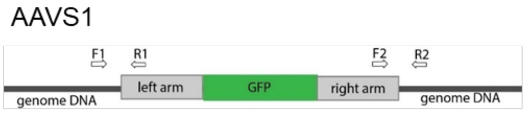

c

Non-precise integration at the junctions		
	Left arm	Right arm
spCas9	Below detection (**60,871 reads**)	Below detection (**11,712 reads**)
miCas9	Below detection (**20,596 reads**)	Below detection (**16,082 reads**)

d

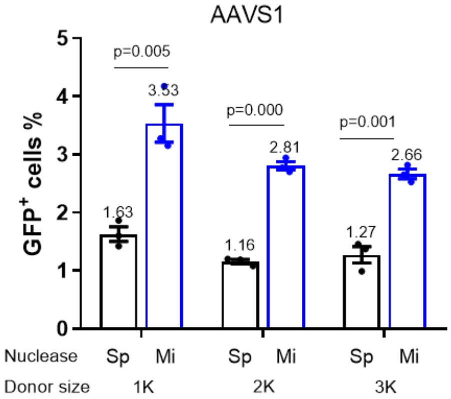

Supplementary Figure 3. **MiCas9 increases KI rates and reduces on-target indel rates in ds-KI applications**. (a) Percentage of GFP+ cells at the ROSA26 locus in different cell types by miCas9 and spCas9 pDNAs. (b - f) Percentage of GFP+ cells and on-target indel rates, estimated by T7EI assays, after GFP ds-KI experiments at ROSA26, AHR, ATF4, TGIF2 and RAD21 loci. Sp: spCas9. Mi: miCas9. HE: Cas9-HE. GE: Cas9-GE. Three independent experiments were performed for each condition. Data are presented as mean ± standard error of means (SEM). Unpaired t test (two-tailed) was used to compare data using GraphPad Prism 8 software (GraphPad Software, Inc., San Diego, CA).

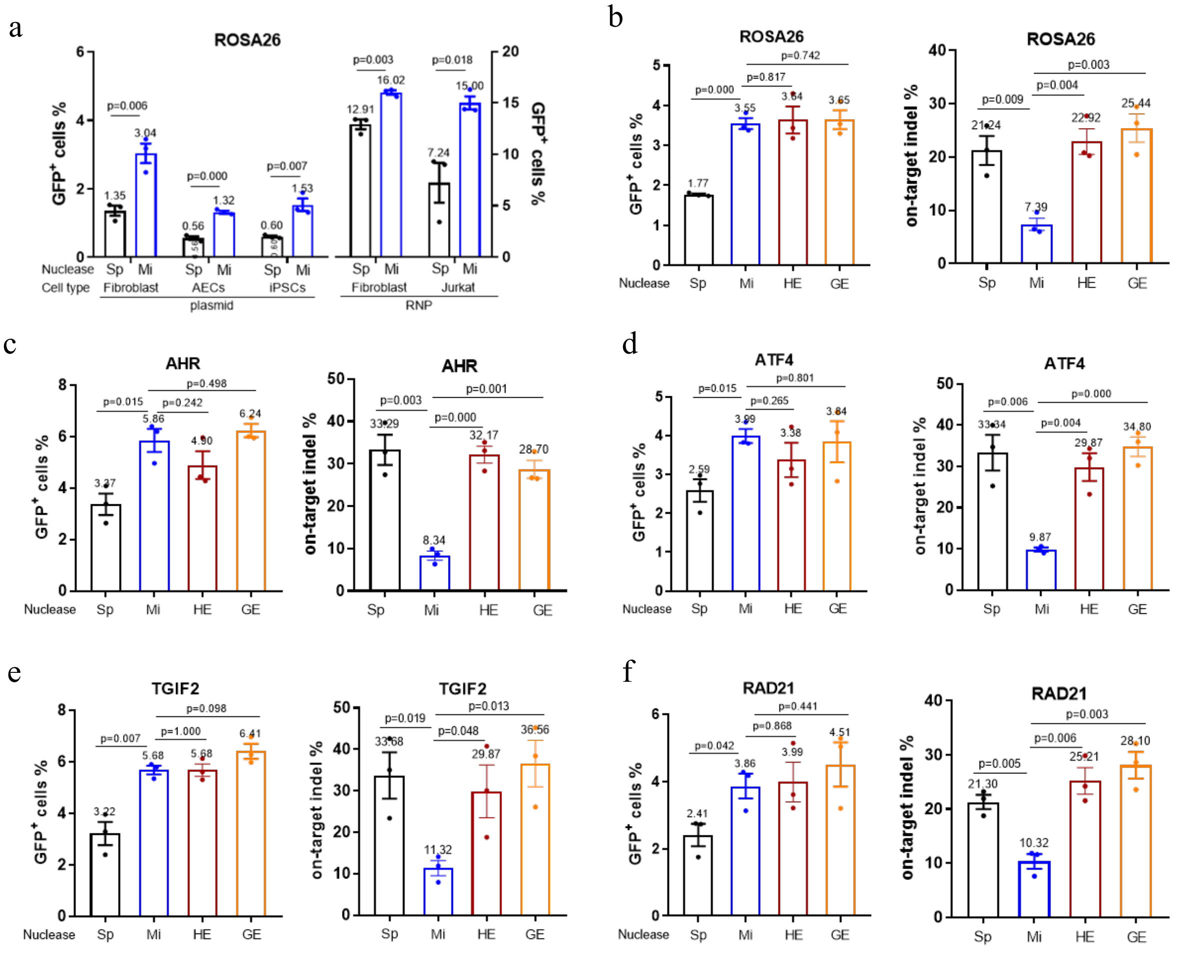

Supplementary Figure 4. Off-target analysis by spCas9 and miCas9. (a) T7EI assay for predicted off-target loci (OT1-OT8) for sg-AAVS1. (b) T7EI assay for predicted off-target loci (OT1-OT5) for sg-Rosa. (c) Off-target and on-target indel rates for sg-EMX1 determined by deep-seq. (d) Off-targets (OT-1 and -2) and on-target indel rates for sg2-VEGFA determined by deep-seq. Sp: spCas9. Mi: miCas9. # Reads: Average amplicon reads per sample. In (c) and (d), three independent experiments were performed for each condition. Data are presented as mean ± standard error of means (SEM). Unpaired t test (two-tailed) was used to compare data using GraphPad Prism 8 software (GraphPad Software, Inc., San Diego, CA).

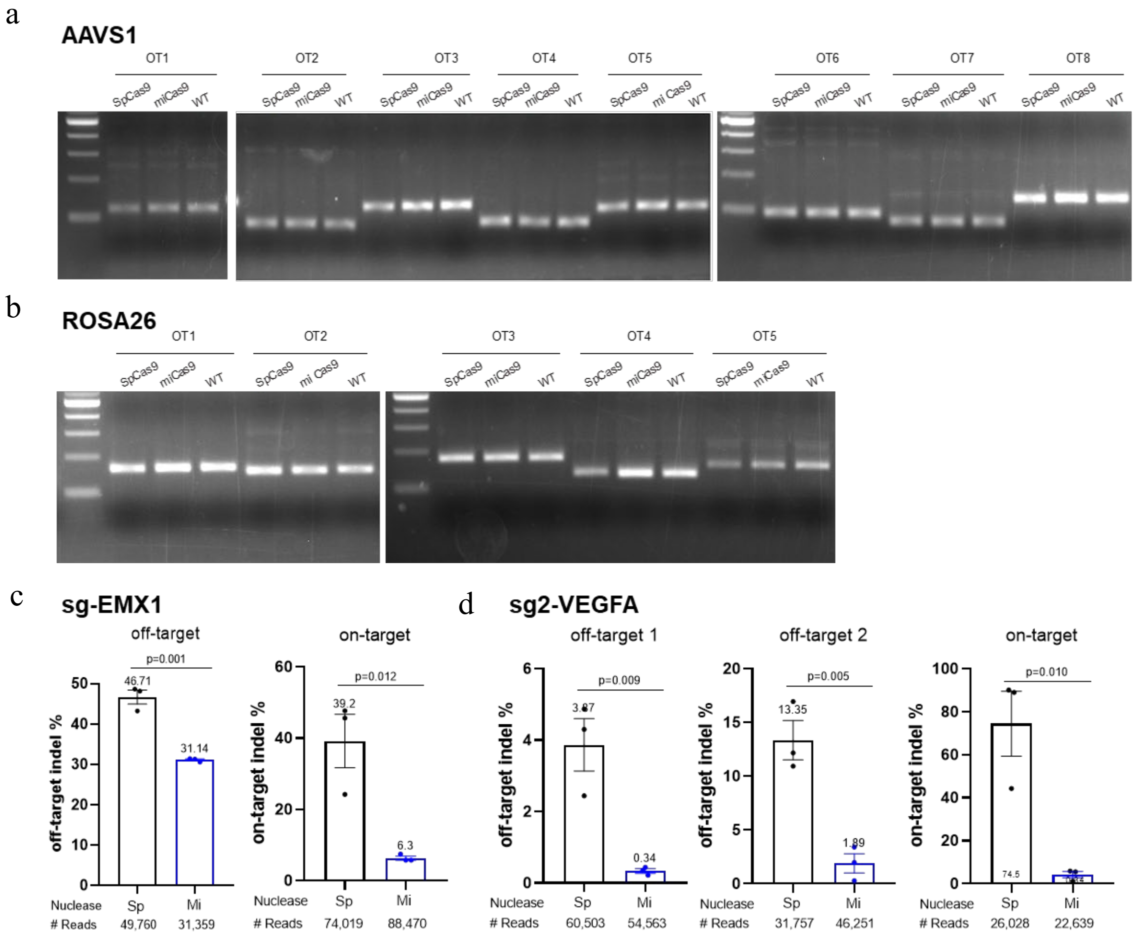

Supplementary Figure 5. Indel rates at Guide-seq predicted potential off-target loci associated with sg1-VEGFA by pDNAs of different nucleases. Sp: spCas9. Mi: miCas9. Hy: hypaCas9. Hi: HiFiCas9. HE: Cas9-HE. GE: Cas9-GE. NC: negative control with non-specific gRNA. # Reads: Average amplicon reads per sample. Three independent experiments were performed for each condition. Data are presented as mean ± standard error of means (SEM). Unpaired t test (two-tailed) was used to compare data using GraphPad Prism 8 software (GraphPad Software, Inc., San Diego, CA).

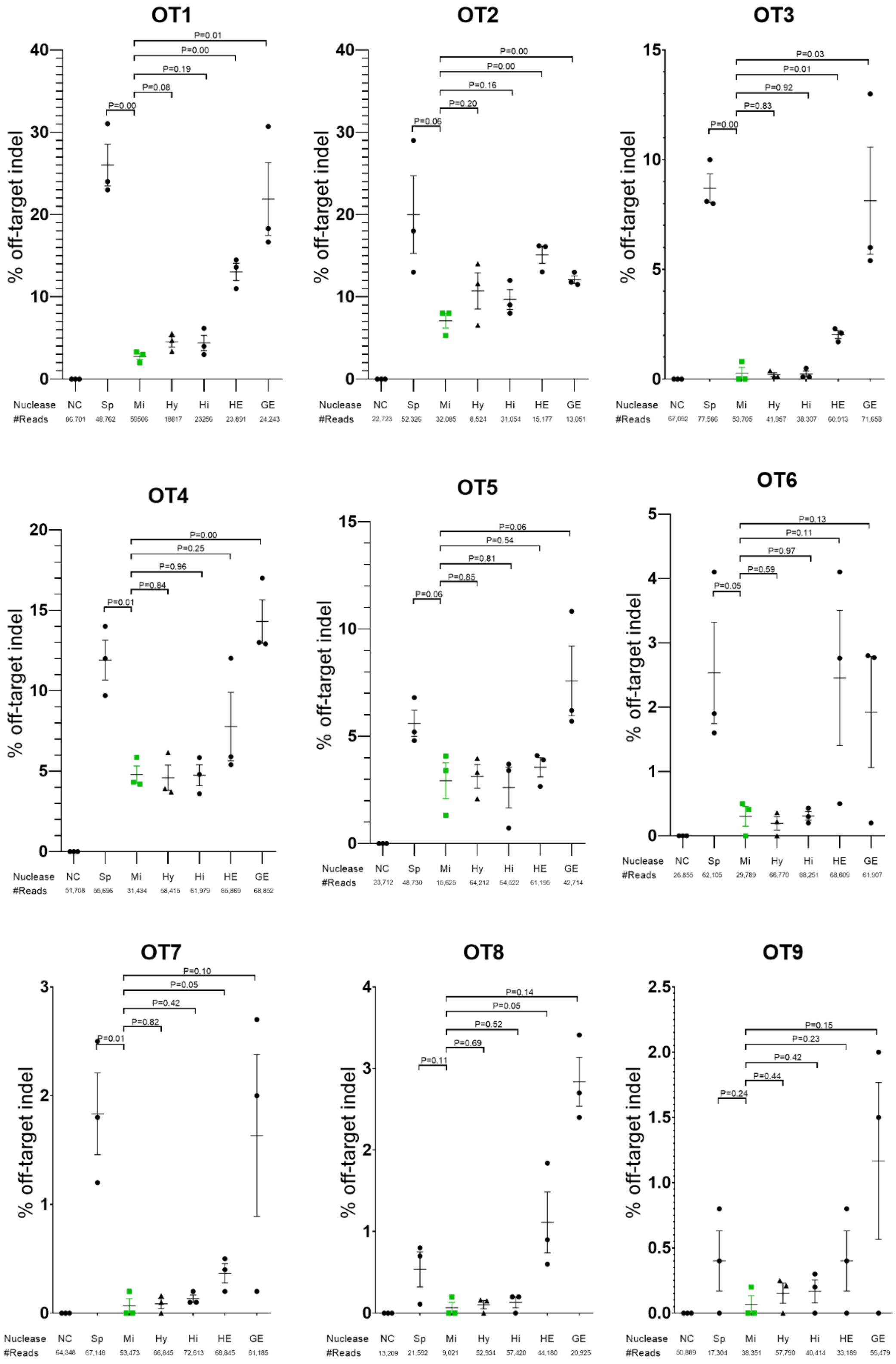
OT1
P=0.01
P=0.00
P=0.19
P=0.08
P=0.00
% off-target indel
Nuclease NC Sp Mi Hy Hi HE GE
#Reads 86,701 48,762 59506 18817 23256 23,891 24,243
OT2
P=0.00
P=0.00
P=0.16
P=0.20
P=0.06
% off-target indel
Nuclease NC Sp Mi Hy Hi HE GE
#Reads 22,723 52,326 32,085 8,524 31,054 15,177 13,051
OT3
P=0.03
P=0.01
P=0.92
P=0.83
P=0.00
% off-target indel
Nuclease NC Sp Mi Hy Hi HE GE
#Reads 67,052 77,586 53,705 41,957 38,307 60,913 71,658
OT4
P=0.00
P=0.25
P=0.96
P=0.84
P=0.01
% off-target indel
Nuclease NC Sp Mi Hy Hi HE GE
#Reads 51,708 55,696 31,434 58,415 61,979 65,869 68,852
OT5
P=0.06
P=0.54
P=0.81
P=0.85
P=0.06
% off-target indel
Nuclease NC Sp Mi Hy Hi HE GE
#Reads 23,712 48,730 15,625 64,212 64,522 61,195 42,714
OT6
P=0.13
P=0.11
P=0.97
P=0.59
P=0.05
% off-target indel
Nuclease NC Sp Mi Hy Hi HE GE
#Reads 26,855 62,105 29,789 66,770 68,251 68,609 61,907
OT7
P=0.10
P=0.05
P=0.42
P=0.82
P=0.01
% off-target indel
Nuclease NC Sp Mi Hy Hi HE GE
#Reads 64,348 67,148 53,473 66,845 72,613 68,845 61,185
OT8
P=0.14
P=0.05
P=0.52
P=0.69
P=0.11
% off-target indel
Nuclease NC Sp Mi Hy Hi HE GE
#Reads 13,209 21,592 9,021 52,934 57,420 44,180 20,925
OT9
P=0.15
P=0.23
P=0.42
P=0.44
P=0.24
% off-target indel
Nuclease NC Sp Mi Hy Hi HE GE
#Reads 50,889 17,304 38,351 57,750 40,414 33,189 56,479

Supplementary Figure 6. Indel rates at Guide-seq predicted potential off-target loci associated with sg-FANCF2 by pDNAs of different nucleases. Sp: spCas9. Mi: miCas9. Hy: hypaCas9. Hi: HiFiCas9. HE: Cas9-HE. GE: Cas9-GE. NC: negative control with non-specific gRNA. # Reads: Average amplicon reads per sample. Three independent experiments were performed for each condition. Data are presented as mean ± standard error of means (SEM). Unpaired t test (two-tailed) was used to compare data using GraphPad Prism 8 software (GraphPad Software, Inc., San Diego, CA).

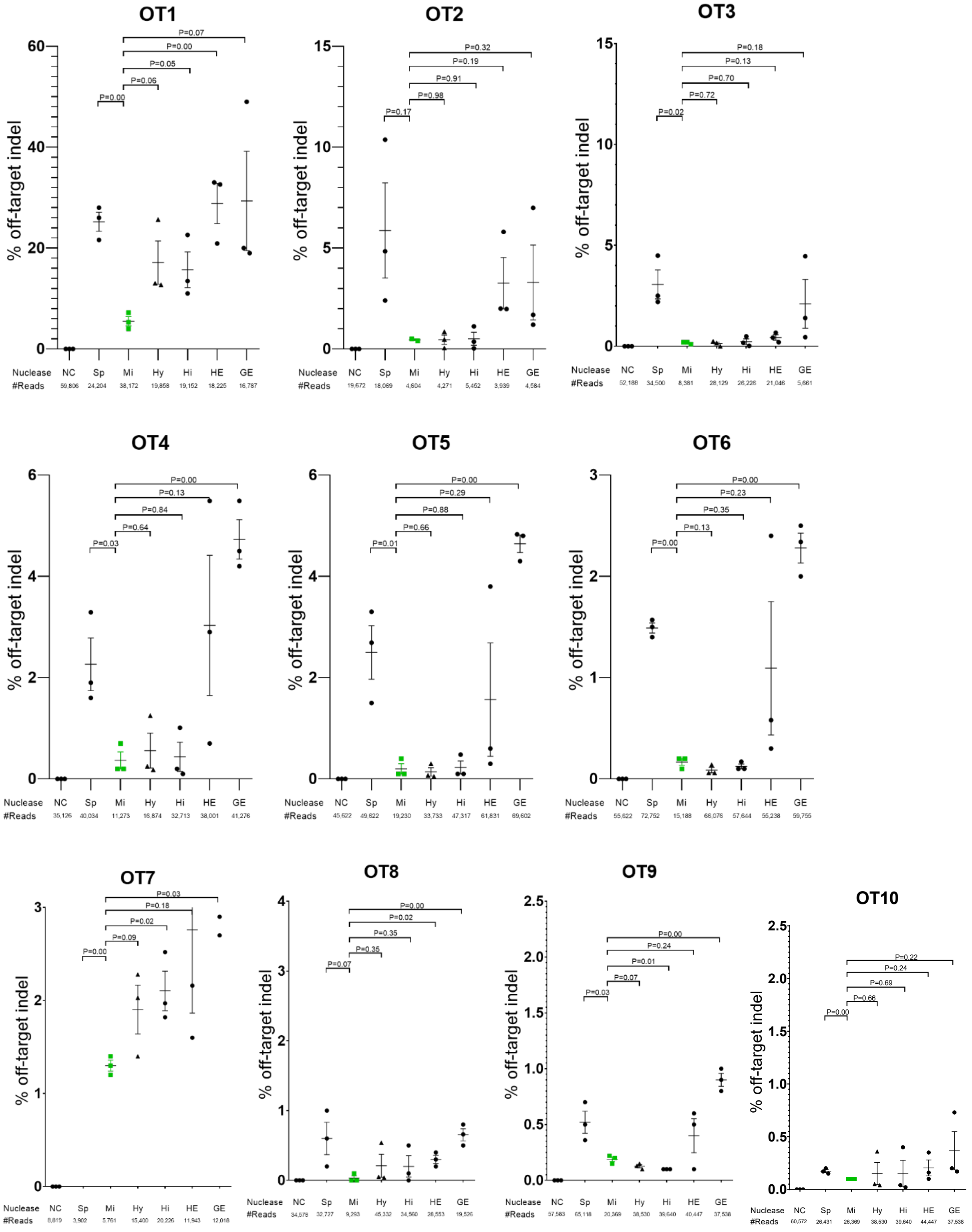
OT1
OT2
OT3
OT4
OT5
OT6
OT7
OT8
OT9
OT10
% off-target indel
Nuclease
#Reads
NC
Sp
Mi
Hy
Hi
HE
GE

Supplementary Figure 7. Effects of miCas9 on ss-PGE applications. (a - c) PGE rates, on-target indel rates, and the Noise/Signal ratio at Mybpc3, NKX2.1 and SOD by spCas9 and miCas9 in human iPSCs. (d) PGE rates, on-target indel rates, and the Noise/Signal ratio at CCR5-del32 by spCas9 and miCas9 in human HSCs. (e - f) PGE rates, on-target indel rates, and Noise/Signal ratio at CCR5-C101 and PSEN1 by different nucleases in human iPSCs. Sp: spCas9. Mi: miCas9. HE: Cas9-HE. GE: Cas9-GE. # Reads: Average amplicon reads per sample. Three independent experiments were performed for each condition. Data are presented as mean ± standard error of means (SEM). Unpaired t test (two-tailed) was used to compare data using GraphPad Prism 8 software (GraphPad Software, Inc., San Diego, CA).

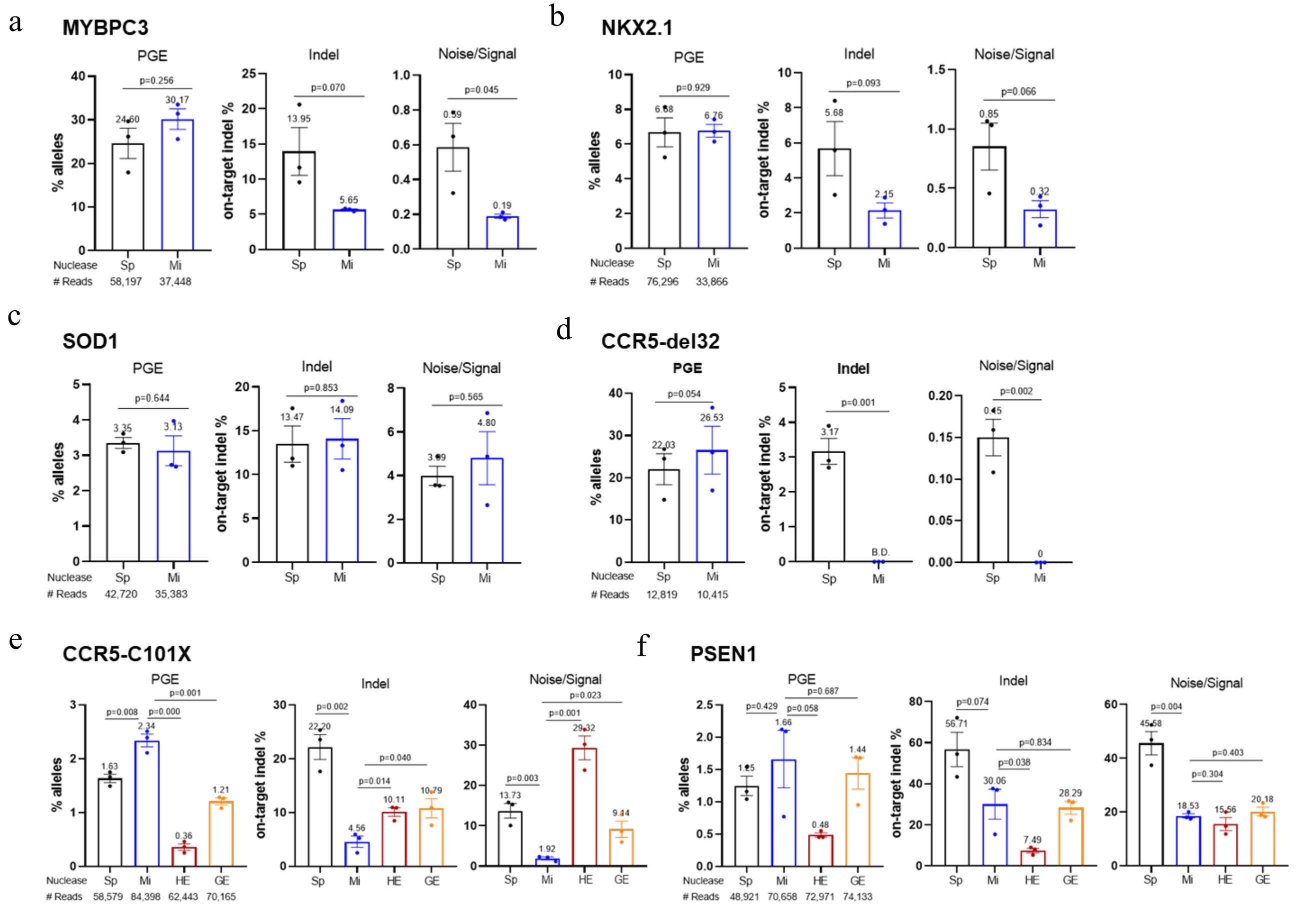

Supplementary Figure 8. Illustration of the mutated Brex27 coding sequence in MiCas9-mut (bottom) in comparison with wild-type Brex27 in miCas9 (top). Red "-" indicates the missing nucleotide in miCas9-mut.

Brex27 coding sequence in miCas9

GCATTGGATTTCCTTAGTAGACTCCCCTTGCCACCTCCGGTGTCCCCCATCTGCACGTTTGTGAGCCCAGCGGCACAGAAGGCATTTCAACCGCCACGGAGTTGCGGG**TAA**

A L D F L S R L P L P P P V S P I C T F V S P A A Q K A F Q P P R S C G *

Mutated Brex27 coding sequence in miCas9-mut

GCATTGGATT-CCTTAGTAGACTCCCCTTGCCACCTCCGGTGTCCCCCATCTGCACGTTTG**TGA**GCCCAGCGGCACAGAAGGCATTTCAACCGCCACGGAGTTGCGGGTAA

A L D S L V D S P C H L R C P P S A R L *

Supplementary Figure 9. Representative FACS plot in the GFP ds-KI experiments. Left panel: negative control, wild-type (WT) Jurkat cells without any nuclease treatment. Middle panel: Jurkat cells treated with spCas9 in the RNP form and GFP donor templates. Right panel: Jurkat cells treated with miCas9 in the RNP form and GFP donor templates. Numbers inside the boxes indicate %GFP+ cells.

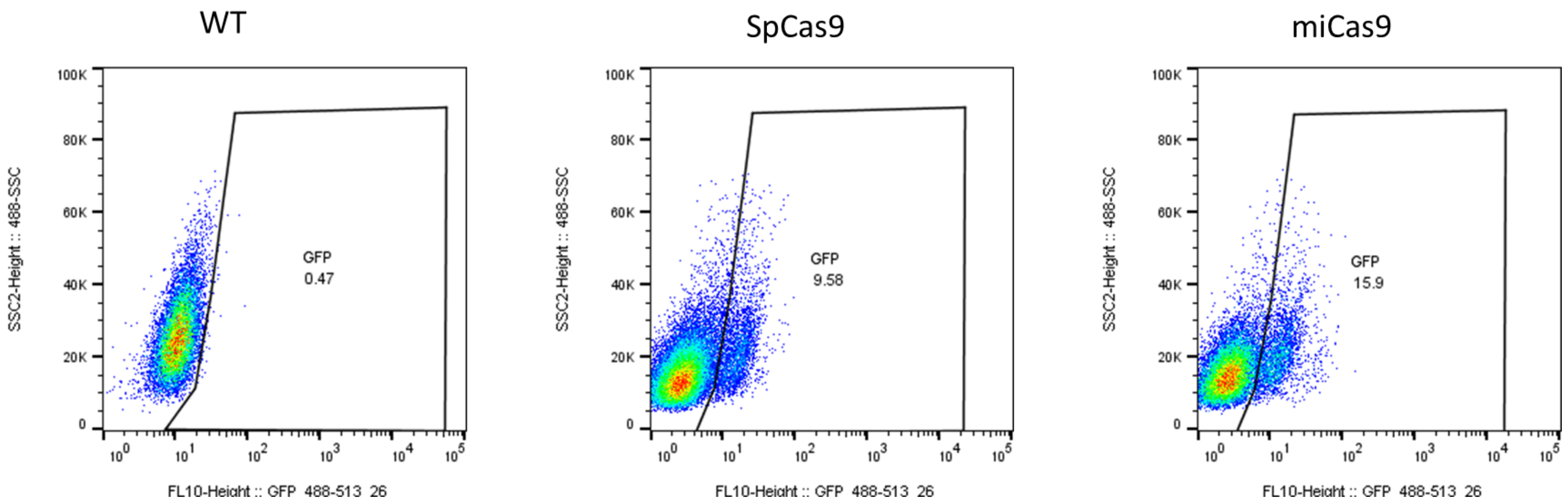

Supplementary Table 1. Information of guide RNAs and donors used in the ds-KI applications.

**ds-KI donor name**	**Target gene**	**Guide RNA name**	**Guide RNA**	**LHA size (bp)**	**RHA size (bp)**	**KI fragment size (bp)**
GFP-donor-1k-AAVS1	AAVS1	sg-AAVS1	GGGGCCACTAGGGACAGGAT TGG	804	837	989
GFP-donor-2k-AAVS1	AAVS1	sg-AAVS1	GGGGCCACTAGGGACAGGAT TGG	804	837	2,469
GFP-donor-3k-AAVS1	AAVS1	sg-AAVS1	GGGGCCACTAGGGACAGGAT TGG	804	837	3,339
GFP-donor-1k-ROSA	ROSA26	sg-ROSA	GTATATAAAACCCCATTTTAT AGG	667	558	989
GFP-donor-1k-RAD	RAD21	sg-RAD	TTCTAGCTCCTTATATAATA TGG	993	1600	989
GFP-donor-1k-TGIF	TGIF2	sg-TGIF	CCAGTAGGCATCTGCCAAGA AGG	799	726	989
GFP-donor-1k-AHR	AHR	sg-AHR	CAAAAACCAGGGTCAAAATT GGG	708	708	989
GFP-donor-1k-ATF	ATF4	sg-ATF	AGAAAAGGGTCCCCTAGTTG AGG	708	711	989

Supplementary Table 2. Guide RNAs used for off-target analysis experiments. Red letters indicate mismatches.

Guide RNA name	Target gene	Off-target #	Guide RNA sequence
Sg-EMX1	EMX1	On-target	GAGTCCGAGCAGAAGAAGAA GGG
		OT1	GAGTTAGAGCAGAAGAAGAA AGG
Sg2-VEGFA	VEGFA	On-target	GACCCCCTCCACCCCGCCTC CGG
		OT1	GGGCCCCTCCACCCCGCCTC TGG
		OT2	GCCCCCACCCACCCCGCCTC TGG
Sg1-VEGFA	VEGFA	On-target	GGTGAGTGAGTGTGTGCGTG TGG
		OT1	AGTGAGTGAGTGTGTGTGTG GGG
		OT2	TGTGGGTGAGTGTGTGCGTG AGG
		OT3	AGAGAGTGAGTGTGTGCATG AGG
		OT4	AGTGAATGAGTGTGTGTGTG TGG
		OT5	TGTGAGTAAGTGTGTGTGTG TGG
		OT6	AGTGTGTGAGTGTGTGCGTG TGG
		OT7	GTTGAGTGAATGTGTGCGTG AGG
		OT8	AGCGAGTGGGTGTGTGCGTG GGG
		OT9	GGTGAGTGAGTGCGTGCGGG TGG
Sg-FANCF2	FANCF2	On-target	GCTGCAGAAGGGATTCCATG AGG
		OT1	GCTGCAGAAGGGATTCCAAG GGG
		OT2	GACGCAGAAGGGACTCCATG GGG
		OT3	GGTACAGAAGGGCTTCCATG AGG
		OT4	GCTGCAAAAAGGATTCCAGG GGG
		OT5	ACTGCAAAATGGATTCCATG GGG
		OT6	GCTGGAGGAAGGATTCCATG GGG
		OT7	TGTGAAGAAGGGTTTCCATG AGG
		OT8	GCCACAGAAGGGATTCTATG AAG
		OT9	GCTGCTGAACAGATTCCATG GGG
		OT10	GCTGCTGAAAGGATTCTATG GGG

Supplementary Table 3. Guide RNAs and ssODNs used in ss-PGE applications. Red letters indicate mutations.

PGE locus	guide RNA sequence	ssODN donor sequence
CCR5-del32	CATACAGTCAGTATCAATTC TGG	ATCTTTACCAGATCTCAAAAAGAAGGTCTTCATTACACCTGCAGCTCTCATTTTCCATACA--------------------------------TTAAAGATAGTCATCTTGGGGCTGGTCCTGCCGCTGCTTGTCATGGTCATCTGCTACTCG
CCR5-C101	TACAATGTGTCAACTCTTGAC AGG	GAGGATGATGAAGAAGATTCCAGAGAAGAAGCCTATAAAATAGAGCCCTGTCAAGAGTTGTCACATTGTATTTCCAAAGTCCCACTGGGCGGCAGCATAGTGAGCCCAGAAGGGGACAGT
B2M	CCAGAAAGAGAGAGTAGCGC GAG	GGGTAGGAGAGACTCACGCTGGATAGCCTCCAGGCCAGAAAGAGAGAGTAGCGCGACGCACAGCTAAGGCCACGGAGCGAGACATCTCGG
NKX2.1	AACAGAAGTACCTGTCGGC CGG	GGAAGCGCCGGGTGCTCTTCTCGCAGGCGCAGGTGTACGAGCTGGAGCGACGCTTCAAGCAACAGAAGTACCTGTCGGCGCCCGAGCTCGAGCACCTGGCCAGCATGATCCACCTGACGCCCACGCAGGTCAAGATCTGGTTCCAGAACCACCGCTACAAAATGAAGCG
EGFR	CTGCGTGATGAGCTGCACGG TGG	ACGTGATGGCCAGCGTGGACAACCCCCACGTGTGCCGCCTGCTGGGCATCTGCCTCACCTCTACAGTCCAACTGATTACCCAGCTCATGCCCTTCGGCTGCCTCCTGGACTATGTCCGGGAACACAAAGACAATATTGGCTCCCAG
HBB	CTTGCCCCACAGGGCAGTAA CGG	TCTGACACAACTGTGTTCACTAGCAACCTCAAACAGACACCATGGTGCATCTGACTCCTGTGGAGAAGTCTGCAGTTACTGCCCTGTGGGGCAAGGTGAACGTGGATGAAGTTGGTGGTGAGGCCCTGGGCAG
SOD1	GTCGCCCTTCAGCACGCACA CGG	TCCGTTGCAGTCCTCGGAACCAGGACCTCGGCGTGGCCTAGCGAGTTATGGCGACGAAGGTCGTGTGCGTGCTGAAGGGCGACGGCCCAGTGCAGGGCATCATCAATTTCGAGCAGAAGGC
CFTR-G542	GAGAAAGACAATATAGTTCT TGG	GCTAGACCAATAATTAGTTATTCACCTTGCTAAAGAAATTCTTGCTCGTTGACCTCCACTCAGTGTGATTCCACCTTCTCAAAGAACTATATTGTCTTTCTCTGCAAACTTGGAGATGTCCTATTACCAAAAATAGAAAATTAGAGAGTCACTTTTAGTAT
PSEN1	GATTTATACAGAACCACCAGG AGG	ATCTGTGTCTCATGCTCACCTTATAGCACCTGTATTTATACAGAACCACCAGGAGAATAGTCACGACAACAATGACACTGATCATGATGGCAGCATTCAG
MYBPC3	GGAGTTTGAGTGTGAAGTAT CGG	GCCCCCTGTGCTCATCACGCGCCCCTTGGAGGACCAGCTGGTGATGGTGGGGCAGCGGGTGGAGTTTGCGAG-----GTATCGGAGGAGGGGGCGCAAGTCAAATGGTGAGTTCCAGAAGCACGGGGCATGGGTGTTGGGGGCAT

Supplementary Table 4. Primers used in the experiments.

Application	Name	Sequence
On-target amplicon in ds-KI experiments at AAVS1 & ChIP assay	AAVS1-F	ATGTGGCTCTGGTTCTGGGT
	AAVS1-R	GGAAGGAGGAGGCCTAAGGA
On-target amplicon in ds-KI experiments at RAD21	RAD21-F	AGTTCTACAGCTTCTTGGTTC
	RAD21-R	ACCTCTGCTCATTATGGAAT
On-target amplicon in ds-KI experiments at Rosa26	ROSA26-F	GTGCTCTGGAATTGAGCCTG
	ROSA26-R	AATTGCTCAAACTCTAAGCC

On-target amplicon in ds-KI experiments at ATF4	ATF4-F	TGATAGAAGAGGTCCGCAAG
	ATF4-R	TATTGGAACACACAGCTACA
On-target amplicon in ds-KI experiments at TFIF2	TGIF2-F	ACTGCACACTCCCATCCCTT
	TGIF2-R	ACGGAAACCCAGGACAGCTG
On-target amplicon in ds-KI experiments at AHR	AHR-F	AGCCAGACCTTTTCCTGAT
	AHR-R	TGCTGACGTCCAACAGTGAC
On-target amplicon in ss-PGE experiments at SOD	SOD-F	GTCCTCGGAACCAGGACCTCG
	SOD-R	GACCCGCTCCTAGCAAAGGTG
On-target amplicon in ss-PGE experiments at PSEN1	PSEN1-F	TGCACTCAATTCTGAATGCT
	PSEN1-R	GTGACAAGAATACCCAACCA
On-target amplicon in ss-PGE experiments at HBB	HBB-F	TGTCTTGTAACCTTGATACC
	HBB-R	GAGCCATCTATTGCTTACAT
On-target amplicon in ss-PGE experiments at EGFR	EGFR-F	TGATGGCCAGCGTGGACAAC
	EGFR-R	ACCAGTTGAGCAGGTACTGGG
On-target amplicon in ss-PGE experiments at MYBPC3	MYBPC3-F	ATGCCCCGTGCTTCTGGAAC
	MYBPC3-R	TCAGGGGAGCCAACCCTCAT
On-target amplicon in ss-PGE experiments at B2M	B2M-F	CATTCGGGCCGAGATGTCTCG
	B2M-R	TCACGGAGCGAGAGAGCACAG
On-target amplicon in ss-PGE experiments at CCR5-C101	CCR5 C101X-F1	TCACTATGCTGCCGCCCAGT
	CCR5 C101X-R1	ACGACAGCCAGGTACCTATCG
On-target amplicon in ss-PGE experiments at CCR5-C101	CCR5 C101X-F2	TCTGACCTGTTTTTCCTTCT
	CCR5 C101X-R2	CAGGTACCTATCGATTGTCA
On-target amplicon in ss-PGE experiments at NKX2.1	NKX2.1-F	CGTCAGGTGGATCATGCTGG
	NKX2.1-R	TGTGCGTTTGTCGCTTACAG
On-target amplicon in ss-PGE experiments at CCR5-delta32	CCR5 DELTA32-F1	CTGTGTTTGCTTTAAAAGCCA
	CCR5 DELTA32-R1	GATTCCCGAGTAGCAGATGA
On-target amplicon in ss-PGE experiments at CCR5-delta32	CCR5 DELTA32-F2	CCCAGGAATCATCTTTACCAG
	CCR5 DELTA32-R2	GATTCCCGAGTAGCAGATGA
On-target amplicon in ss-PGE experiments at CFTR-G542	CFTR-G542X-F	GTGCCTTTCAAATTCAGATT
	CFTR-G542X-R	TTACAGCAAATGCTTGCTAG
Off-target amplicon of sg-Rosa26 at OT1	ROSA-OT-F1	CAGCTTGGACTATTTGGTCA
	ROSA-OT-R1	TACCCTATGCTGCTACTCAC
Off-target amplicon of sg-Rosa26 at OT2	ROSA-OT-F2	AGAGCTGTCCTTAGTTCTCC
	ROSA-OT-R2	GAAGCCACTGTGGAGAGTCA
Off-target amplicon of sg-Rosa26 at OT3	ROSA-OT-F3	GCTCCCAGTGGATACATGAA
	ROSA-OT-R3	TGCATAGCCCCCTTGCATTA
Off-target amplicon of sg-Rosa26 at OT4	ROSA-OT-F4	GGATTTCTGGAAGGTTCTGC
	ROSA-OT-R4	ACCACAGAAAAACACAGAGTC
Off-target amplicon of sg-Rosa26 at OT5	ROSA-OT-F5	CCTTCATCGACCTCTGACTC
	ROSA-OT-R5	GCTCATGCAGTAGAGGCTAG
Off-target amplicon of sg-AAVS1 at OT1	AAVS1-OT-F1	GCTCCACTGTGAGAAGCCTT
	AAVS1-OT-R1	CAGAGCTGGGTACAGTGAGG
Off-target amplicon of sg-AAVS1 at OT2	AAVS1-OT-F2	GTGATGGGGTTGGCACTGAT

	AAVS1-OT-R2	GGGACAGCTATACCTGCTCC
Off-target amplicon of sg-AAVS1 at OT3	AAVS1-OT-F3	ACAGAGTCCCACAACGATGG
	AAVS1-OT-R3	AACTCCAGGGGTACAGGAGG
Off-target amplicon of sg-AAVS1 at OT4	AAVS1-OT-F4	CTCAGGCCACATTCCGCA
	AAVS1-OT-R4	TAAGCTGACCTGCTCCGTTG
Off-target amplicon of sg-AAVS1 at OT5	AAVS1-OT-F5	GGATGCTGATGGGCTGTTCT
	AAVS1-OT-R5	CAGGTGGAGGCTGCCATTTA
Off-target amplicon of sg-AAVS1 at OT6	AAVS1-OT-F6	CATAGGACCAGCCAGCAGAG
	AAVS1-OT-R6	AGGCAAACCAAGTACACTTTGT
Off-target amplicon of sg-AAVS1 at OT7	AAVS1-OT-F7	CACCAGTGGAATCAGGGGTC
	AAVS1-OT-R7	ACATGGCTGACTGCTCCATC
Off-target amplicon of sg-AAVS1 at OT8	AAVS1-OT-F8	AACTCTCTAACAGCTGGGGC
	AAVS1-OT-R8	GAGCTGCTCCTTCTTGGGAA
Off-target amplicon of sg-FANCF2 at OT10	F2-OT10-F	GAATGCATTGCTTAATGTGC
	F2-OT10-R	ATACTTAACTCCCCTGACTG
Off-target amplicon of sg-FANCF2 at OT9	F2-OT9-F	TGCTGCAGGGATCTCTAACA
	F2-OT9-R	TATTCCTCACCGTGAACCTA
Off-target amplicon of sg-FANCF2 at OT8	F2-OT8-F	CTTCTAACAGTGAGTGGGTC
	F2-OT8-R	CTTCTTAGCCCGAGTAAGAC
Off-target amplicon of sg-FANCF2 at OT7	F2-OT7-F	AGCCTGGGCAACAGAGCAAG
	F2-OT7-R	CTGTGTACAAAGCCCAGGGT
Off-target amplicon of sg-FANCF2 at OT6	F2-OT6-F	GTGAACCCACCAAAGGCGGA
	F2-OT6-R	AGGAGACACTTCTGTGGGGC
Off-target amplicon of sg-FANCF2 at OT5	F2-OT5-F	TGATAGAGGCATCTGCTACT
	F2-OT5-R	CTGGGAATTGTCTCATCAG
Off-target amplicon of sg-FANCF2 at OT4	F2-OT4-F	TACTGTCCACCATCTCAGAG
	F2-OT4-R	TCAGAGAGTAAGGACCTGGA
Off-target amplicon of sg-FANCF2 at OT3	F2-OT3-F	TAAGGGGGACCTTGAAATAT
	F2-OT3-R	AGTCCCACAGTTCATAAACAA
Off-target amplicon of sg-FANCF2 at OT2	F2-OT2-F	CAGCATGGGGGCAAAGAGCA
	F2-OT2-R	TCCAGGGCCCTATCTCAGCT
Off-target amplicon of sg-FANCF2 at OT1	F2-OT1-F	GCCCTACTGACTGACAGAGG
	F2-OT1-R	TCTCCTGTTCTGGCTCCCTT
Off-target amplicon of sg-EMX1	EMX1-OT1-F	GTGGGGAGATTTGCATCTGTGGAGG
	EMX1-OT1-R	GCTTTTATACCATCTTGGGGTTACAG
Off-target amplicon of sg1-VEGFA at OT1	VEGFA1-OT1-F	TATCGCTCATTTCCTACGGC
	VEGFA1-OT1-R	GCAGTGAGGAGGTGGTTCTT
Off-target amplicon of sg1-VEGFA at OT2	VEGFA1-OT2-F	TTCAGGGTGTGCAATGTGA
	VEGFA1-OT2-R	TTCTGGGAATCTAATGTATGGCA
Off-target amplicon of sg1-VEGFA at OT3	VEGFA1-OT3-F	TTGGATCATACGGCCGGTTT
	VEGFA1-OT3-R	AAAGTTTCACATGGTTGCGG
Off-target amplicon of sg1-VEGFA at OT4	VEGFA1-OT4-F	GTTCACTCGGCTACAGGGAG
	VEGFA1-OT4-R	ATAAGGGGCAAGTTCTGGGC

Off-target amplicon of sg1-VEGFA at OT5	VEGFA1-OT5-F	TTGCTGTGTCTTCCTTCTGC
	VEGFA1-OT5-R	AGCTGTATGTGAGTCCCTGA
Off-target amplicon of sg1-VEGFA at OT6	VEGFA1-OT6-F	CCCCATTGCCTAGAAGAGTCA
	VEGFA1-OT6-R	AACCCTTGGGAATCTATCTTGAA
Off-target amplicon of sg1-VEGFA at OT7	VEGFA1-OT7-F	AGACCCAGTTCCAAGCCAG
	VEGFA1-OT7-R	TCTCCGGAAGTGCCTTGC
Off-target amplicon of sg1-VEGFA at OT8	VEGFA1-OT8-F	AGCACCCTTGACGTCTGG
	VEGFA1-OT8-R	AGCTCACCTTCCAGTTCCG
Off-target amplicon of sg1-VEGFA at OT9	VEGFA1-OT9-F	GTCTCCAGGCCACAGAGTAG
	VEGFA1-OT9-R	GCACCCCAACACCTACATCT
Off-target amplicon of sg2-VEGFA at OT1	VEGFA2-OT1-F	CAAGATGTGCACTTGGGCTA
	VEGFA2-OT1-R	GCAGCCTATTGTCTCCTGGT
Off-target amplicon of sg2-VEGFA at OT2	VEGFA2-OT2-F	GTTGCTCTTTGTTGAGAGGG
	VEGFA2-OT2-R	TCTTGCTCTGACCTTGTTTG
Nest PCR for junctions of AAVS1	JUNCTION-F1	GAGCTCTACTGGCTTCTGCG
	JUNCTION-F2	CAGGCCAAGTAGGTGGCCTG
	JUNCTION-F3	TGCATTCTAGTTGTGGTTTG
	JUNCTION-R1	GCCGTAGAGGTGACCCAGGC
	JUNCTION-R2	CTTCCTCGTCCACCATCTCA
	JUNCTION-R3	AGCTCGACCAGGATGGGCAC
