# Supplementary material for: MiCas9 increases large size gene knock-in rates and reduces undesirable on-target and off-target indel edits
Source: Nat Commun. 2020 Nov 27;11:6082. doi: 10.1038/s41467-020-19842-2 (PMC7695827; doi:10.1038/s41467-020-19842-2)
Supplement: Supplementary file 2 — Reporting Summary [file 41467_2020_19842_MOESM2_ESM.pdf]

nature research

Corresponding author(s): Y. Eugene Chen, Jifeng Zhang, Jie Xu

Last updated by author(s): Oct 6, 2020

Reporting Summary

Nature Research wishes to improve the reproducibility of the work that we publish. This form provides structure for consistency and transparency in reporting. For further information on Nature Research policies, see our Editorial Policies and the Editorial Policy Checklist.

Statistics

For all statistical analyses, confirm that the following items are present in the figure legend, table legend, main text, or Methods section.

n/a	Confirmed	
☐	☒	The exact sample size (n) for each experimental group/condition, given as a discrete number and unit of measurement
☐	☒	A statement on whether measurements were taken from distinct samples or whether the same sample was measured repeatedly
☐	☒	The statistical test(s) used AND whether they are one- or two-sided<br>*Only common tests should be described solely by name; describe more complex techniques in the Methods section.*
☒	☐	A description of all covariates tested
☒	☐	A description of any assumptions or corrections, such as tests of normality and adjustment for multiple comparisons
☐	☒	A full description of the statistical parameters including central tendency (e.g. means) or other basic estimates (e.g. regression coefficient) AND variation (e.g. standard deviation) or associated estimates of uncertainty (e.g. confidence intervals)
☐	☒	For null hypothesis testing, the test statistic (e.g. F, t, r) with confidence intervals, effect sizes, degrees of freedom and P value noted<br>*Give P values as exact values whenever suitable.*
☒	☐	For Bayesian analysis, information on the choice of priors and Markov chain Monte Carlo settings
☒	☐	For hierarchical and complex designs, identification of the appropriate level for tests and full reporting of outcomes
☒	☐	Estimates of effect sizes (e.g. Cohen's d, Pearson's r), indicating how they were calculated

Our web collection on statistics for biologists contains articles on many of the points above.

Software and code

Policy information about availability of computer code

Data collection	BD FACS Diva Software, version 8.
Data analysis	FlowJo (v10), CRISPResso2 (https://crispresso.pinellolab.partners.org/submission), Blastn (https://blast.ncbi.nlm.nih.gov/Blast.cgi).

For manuscripts utilizing custom algorithms or software that are central to the research but not yet described in published literature, software must be made available to editors and reviewers. We strongly encourage code deposition in a community repository (e.g. GitHub). See the Nature Research guidelines for submitting code & software for further information.

Data

Policy information about availability of data

All manuscripts must include a data availability statement. This statement should provide the following information, where applicable:

- Accession codes, unique identifiers, or web links for publicly available datasets
- A list of figures that have associated raw data
- A description of any restrictions on data availability

All targeted amplicon sequencing data have been deposited at the Sequence Read Archive BioProject with accession number PRJNA615686, which contains fastq files for generating the main and supplementary figures. Other relevant data are available from the corresponding authors on request.

Field-specific reporting

Life sciences study design

All studies must disclose on these points even when the disclosure is negative.

Sample size	Sample sizes were determined based on prior experience and widely used sizes in similar publications.
Data exclusions	There were no datasets excluded from analysis in this work.
Replication	All experiments were performed in triplicates or more to establish reproducibility, except for the off-target analysis of GUIDE-seq predicted loci, for which 3 replicates were performed. Altogether, the level of reproducibility in these studies were very high.
Randomization	There were no live animals or human participants in this study for randomization. For in vitro study, specific cell types were chosen to demonstrate the applicability in these cell types, therefore the choice of cell types are not randomized.
Blinding	Experiments, data collection and analysis were carried out by the same person(s), therefore no blinding was used.

Reporting for specific materials, systems and methods

We require information from authors about some types of materials, experimental systems and methods used in many studies. Here, indicate whether each material, system or method listed is relevant to your study. If you are not sure if a list item applies to your research, read the appropriate section before selecting a response.

Materials & experimental systems

n/a	Involved in the study	
☐	☒	Antibodies
☐	☒	Eukaryotic cell lines
☒	☐	Palaeontology and archaeology
☒	☐	Animals and other organisms
☒	☐	Human research participants
☒	☐	Clinical data
☒	☐	Dual use research of concern

Methods

n/a	Involved in the study	
☒	☐	ChIP-seq
☐	☒	Flow cytometry
☒	☐	MRI-based neuroimaging

Antibodies

Antibodies used	The RAD51 antibodies were purchased from Novus Biologicals, Cat# NB100-148, and from Abclonal, Cat# A6268. The Cas9 antibody was purchased from Abclonal, Cat# A14997.
Validation	According to the manufacturer Novus Biologicals, the RAD51 antibody NB100-148 was validated in human and mouse in different applications: including ChIP, ICC/IF, IHC, IHC-P, In vitro, Western blot.<br><br>According to the manufacturer Abcolonal, the RAD51 antibody A6268 was validated in human, mouse and rat in Western blot.<br><br>According to the manufacturer Abcolonal, the Cas9 antibody was validated in human cells in 2 applications: Western blot and Immunofluorescence.

Eukaryotic cell lines

Policy information about cell lines

Cell line source(s)	Human fibroblast cells (Cat#CRL2522), human iPSC cells (Cat#ACS-1030), human CD34+ hematopoietic stem cells (Cat#PCS-800-012), human airway epithelial cells (Cat#PCS-300-010), and human Jurkat cells (Cat# TIB-152, ATCC) were acquired from American Type Culture Collection (ATCC, Manassas, VA). Human Ad293 cells (Cat#240085) was purchased from Agilent (Santa Clara, CA).
Authentication	Cells from ATCC were authenticated by the ATCC using morphology, karyotyping, and PCR based approaches to confirm the identity and to rule out both intra- and interspecies contamination. The Ad293 cells were authenticated by the lab based on morphology observations.
Mycoplasma contamination	All cell lines have been tested for mycoplasma contamination and resulted negative.
Commonly misidentified lines (See ICLAC register)	None of the cells used in this study are listed in the ICLAC register.

Flow Cytometry

Plots

Confirm that:

- [x] The axis labels state the marker and fluorochrome used (e.g. CD4-FITC).
- [x] The axis scales are clearly visible. Include numbers along axes only for bottom left plot of group (a 'group' is an analysis of identical markers).
- [x] All plots are contour plots with outliers or pseudocolor plots.
- [x] A numerical value for number of cells or percentage (with statistics) is provided.

Methodology

Sample preparation	5x10^5 cells were resuspended in 300 µL PBS with 2 % FBS, filtered with 70 µm nylon strainer, and then analyzed for GFP positive cell population.
Instrument	MoFlo Astrios EQs Sorter (Model# B52102, from Beckman Coulter, Indianapolis, IN)
Software	Data collection: BD FACS Diva Software, version 8.<br>Data analysis: FlowJo (v10).
Cell population abundance	Cell numbers were sufficient and normalized for each analysis.
Gating strategy	Cells were gated base on FSC/SSC, doublet discrimination, live/dead, and then by GFP expression.

- [x] Tick this box to confirm that a figure exemplifying the gating strategy is provided in the Supplementary Information.
